# Radiographic predictors of peritumoral brain edema in intracranial meningiomas: a review of current controversies and illustrative cases

**DOI:** 10.1186/s41016-024-00383-2

**Published:** 2024-10-28

**Authors:** Darko Orešković, Andrea Blažević, Anđelo Kaštelančić, Ivan Konstantinović, Marin Lakić, Filip Murn, Marko Puljiz, Martina Štenger, Pia Barač, Darko Chudy, Tonko Marinović

**Affiliations:** 1 Department of Neurosurgery, Clinical Hospital Dubrava, Zagreb, Croatia; 2 Department of Neurosurgery, University Hospital Center Split, Split, Croatia; 3 Department of Neurosurgery, General Hospital Dubrovnik, Dubrovnik, Croatia; 4 Department of Radiology, Children’s Hospital Zagreb, Zagreb, Croatia; 5 Department of Radiology, Clinical Hospital Dubrava, Zagreb, Croatia; 6 Department of Neurosurgery, Children’s Hospital Zagreb, Zagreb, Croatia; 7 School of Medicine, University of Zagreb, Zagreb, Croatia; 8 Medicine of Sports and Exercise, Faculty of Kinesiology, University of Zagreb, Zagreb, Croatia

**Keywords:** Meningioma, Peritumoral brain edema, MRI

## Abstract

Meningiomas are among the most common primary tumors of the central nervous system. In the past several decades, many researchers have emphasized the importance of radiographic findings and their possible role in predicting the various aspects of the meningioma biology. One of the factors most commonly analyzed with respect to the lesions’ clinical behavior is peritumoral brain edema (PTBE), not only one of the most common signs associated with meningiomas, but also a significant clinical problem. Radiographic predictors of PTBE are usually noted as being the size of the tumor, its location, irregular margins, heterogeneity, and the peritumoral arachnoid plane with its pial vascular recruitment. Here, we review the available literature on the topic of these radiographic predictors of PTBE formation, we analyze the methodology of the research conducted, and we highlight the many controversies still present. Indeed, the evidence about PTBE pathogenesis, predictive factors, and clinical significance still seems to be mostly inconclusive, despite intense research in the area. We believe that by highlighting the many inconsistencies in the methodology used, we can showcase how little is actually known about the pathogenesis of PTBE, which in turn has important clinical implications. Additionally, we provide several MR images of intracranial meningiomas from our own practice which, we believe, showcase the unpredictable nature of PTBE, and demonstrate vividly the topics we discuss.

**Supplementary Information:**

The online version contains supplementary material available at 10.1186/s41016-024-00383-2.

## Background

Meningiomas are among the most common primary tumors of the central nervous system. They are present in 1% of the general population, with an incidence of around 7.8 / 100,000 [1, 2]. According to the current WHO classification [3], roughly 80% of meningiomas are considered grade 1 lesions, with a 10-year overall survival rate of up to 90%. WHO grade 2 lesions constitute around 20% of all meningiomas and have a 10-year overall survival rate of around 53%. Meningiomas which are diagnosed as WHO grade 3 are found in only 2–3% of patients and have a 10-year overall survival rate of 0% [1, 2]. Complete surgical excision remains the primary treatment modality for all types of meningiomas, with radiation therapy being a supplementary treatment option. There are no chemotherapeutic agents currently in routine use for the treatment of meningioma patients [4].

The analysis of radiographic imaging, alongside the medical history and the clinical examination of the patient, is the foundation on which a decision is made about the surgical removal of the meningioma [5, 6]. It is therefore of crucial importance that radiographic findings are reliable with regard to the biology of the neoplasm. And indeed, in the past several decades, many researchers have emphasized the importance of radiographic analysis and its possible role in predicting the various aspects of the meningioma biology [7–12]. And one of the factors most commonly analyzed with respect to the lesions’ clinical behavior is peritumoral brain edema (PTBE).

PTBE is one of the most common signs associated with meningiomas, found in up to 70% of all cases. It occurs in the surrounding brain tissue, which displays an increase in the water content [13], usually thought to be visualized on a MR scan as a T2 hyperintensity [14, 15]. Of note is that the severity of PTBE is recognized to be mostly an investigator-dependent assessment, and not an objective finding [16]. PTBE seems to aggravate the disease symptoms [16–18] and is often connected to higher morbidity, longer hospital stays, and more medical support needed [16, 18–22]. PTBE has also been reported to complicate surgical management as well as impact the safety of radiosurgery [13, 23, 24]. Indeed, addressing PTBE is recognized as being a crucial part in the treatment of patients with brain tumors [13]. Pathophysiological mechanisms which cause PTBE are usually thought to be primarily vasogenic and cytotoxic in nature. Vasogenic edema occurs due to extracellular fluid accumulation in the brain parenchyma, caused by the disruption in the blood–brain barrier and leading to serum protein build-up in the extracellular space. Cytotoxic edema forms after an injury to glial, epithelial, and/or neuronal cells, which causes cellular swelling and a complex injury cascade [25, 26]. In meningiomas specifically, four main theories have emerged to explain the pathogenesis of PTBE. These are the brain parenchyma compression theory, secretory-excretory theory, venous compression theory, and the hydrodynamic theory [27, 28]. Of note is however that the exact mechanisms which lead to PTBE formation are still largely unknown, with all proposed theories likely having some role in the pathogenesis [27, 29], and with novel theories emerging [15]. Of note, there are several other non-radiographic predictors which are known to possibly influence the extent of PTBE. However, these are beyond the scope of this review and are discussed at length elsewhere [29].

Predictors of PTBE which can be seen on a MR scan are usually noted as being the size of the tumor, its location, irregular margins, heterogeneity, and the peritumoral arachnoid plane with its pial vascular recruitment [27, 30]. However, the exact relationship of all these factors to PTBE formation and extent remains elusive. Here, we review the available literature on the topic, analyze the methodology of the research conducted, and highlight the many controversies still present. With this, we hope to draw attention to the fact that PTBE is not only one of the most common signs in patients with meningiomas, but also one of the most poorly understood. Indeed, the evidence about PTBE pathogenesis, predictive factors, and clinical significance still seems to be mostly inconclusive, despite the intense research in the area. This, in turn, has important clinical implications. Additionally, we provide several MR images of intracranial meningiomas from our own practice which, we believe, showcase the unpredictable nature of meningioma PTBE, and demonstrate vividly the topics we discuss.

A MEDLINE search via the PubMed interface was performed for all articles using the key words: “meningioma” and “peritumoral brain edema.” Search result abstracts were reviewed for pertinent articles in English, including clinical trials and case series, in which a statistical analysis was performed correlating PTBE and the aforementioned radiographic (CT and/or MR) findings. Reference sections of reviewed publications were searched for additional research not identified by the original MEDLINE search. Twenty-two articles were identified which satisfied the search criteria.

### Size and location

Larger meningiomas are thought to be higher-grade lesions more frequently [12, 31], have higher recurrence rates [32] as well as a higher likelihood of growth [33]. The meningioma location has also often been connected to a higher-grade neoplasm [31, 34]. Similarly, both meningioma size and its location have been studied with regard to their connection to PTBE.

For example, there have been several reports of both size and location of the meningioma being independent factors influencing the extent of PTBE [35–39] (Image 1). There have also been reports of PTBE being only dependent on the tumor location, and not on its size [14, 40]. On the other hand, some researchers found that tumor volume was important in PTBE development [41], while the location of the lesion did not seem to be connected [16, 42]. In contrast to these findings, many researchers did not find the correlation between PTBE and either the size of the tumor [43–48] or its location [43–49] (Image 2). Uncertainties also remain whether the size or location of the tumor is connected to the peritumoral blood supply of the meningioma. Indeed, some researchers claim that large skull base meningiomas are more commonly associated with a significant pial blood supply to the lesion [50], while others claim that neither size nor location of the tumor is connected to the extent of pial neo-vasculature [51].

Such different conclusions are not surprising since in the analysis of the connection between PTBE and tumor volume or location there exists a significant discrepancy in the methodology used. As mentioned earlier, the extent of PTBE itself (or its severity) has proven to be challenging to objectify. Some authors for example dichotomize the presence of PTBE into two groups, namely if it was present or absent [14, 36, 43, 45, 48, 52]. In contrast, other researchers calculated the exact PTBE volume using various software and utilizing the formula for a spheroid (4/3πabc), where a,b, and c were the largest diameters of PTBE in their respective planes. These results were then sometimes analyzed as a scalar value [35, 44, 46, 47], and other times categorized into two [45], three [16, 37, 40], or four [8, 51, 53] distinct groups based on the mean PTBE size. Some authors even classified PTBE based on both its size as well as its extension into the surrounding white matter [41], while others only estimated its size comparing it to the size of the meningioma [39].

Similarly, when measuring the size of the tumor, researchers usually calculated the maximum diameter of the tumor in three planes and then calculated the volume of a spheroid [14, 35, 39, 41, 42, 44–49, 54]. Some authors however opted for different options, ranging from simply noting the largest diameter of the lesion [36, 43, 51] to measurement of the tumor volume using various computer programs [16, 37].

The exact tumor location within the cranial vault has also often been difficult to categorize, due primarily to the many possible ways of differentiating the complex cranial anatomy. Sometimes researchers opted for a simple dichotomy between skull base and non-skull base meningiomas [14, 16, 37, 48, 51]. Other times more categories were added to the meningioma location, analyzing four [36], five [41], six [45], seven [44, 46, 52], eight [43], nine [40], and even thirteen [35] distinct groups of meningiomas based on their location. Such discrepancies are detrimental to the investigation of the connection of the tumor location and PTBE since very different tumors are grouped in many different ways, thus obfuscating the possible correlation. An interesting example of the importance of uniform methodology is an article by Lee et al., which reported that the volume of PTBE did not correlate with the volume of the tumor. However when dichotomized simply into meningiomas with PTBE and without it, larger tumors did in fact exhibit significantly larger peritumoral brain edema [46]. Choosing one calculation method over the other can thus clearly influence the obtained results as well as the subsequent conclusions.

### Peritumoral arachnoid plane

Despite meningiomas being considered extra-axial tumors with a layer of a watertight arachnoid membrane between themselves and the brain, there is an increasing awareness that this interface can often be disrupted. Such a disruption can enable the tumor to adhere to the brain parenchyma, leading to the proliferation of blood vessels between the brain and meningioma. It can also lead to difficulties in intraoperative separation of the two [8] and has even been suggested as possibly predictive of a higher-grade meningioma, higher recurrence rates, and several perioperative complications [10, 32, 48, 50–52, 55, 56].

The disrupted arachnoid membrane is thought to be visualized on MRI as an absence of a T2 hyperintense peritumoral rim, while the aforementioned peritumoral neovascularization is thought to be visualized with the so-called “flow voids,” areas of T2 hypointensity [51] (Image 3). And it is this absence of an obvious arachnoid plane which seems to be frequently connected to a large PTBE [8, 14, 16, 35, 43, 47, 51, 52, 54]. However, there have been several reports where a loss of a peritumoral hyperintense rim could not be correlated to a more pronounced PTBE (Image 4), and where a large PTBE was observed around meningiomas despite a significant peritumoral plane [37, 46, 53] (Image 5). Also, whether or not larger meningiomas disrupt the arachnoid membrane around them more often is still unresolved, with some reports claiming that it does [52], and others claiming otherwise [53].

Such a discrepancy in the obtained results can be, at least in part, explained by different methodologies of the research. So for example, not including researchers who analyzed only CT scans [54], most of the cited articles list an arachnoid plane or CSF cleft as being simply present or absent [8, 16, 35, 47, 52]. Some authors use an additional characteristic of an arachnoid plane, namely they consider it present only if it exhibits an additional hypointensity on T1-weighted image [37, 43, 46]. In some articles, the investigators considered the arachnoid plane present if T2 hyperintensity could be seen in any image and in any direction [14], while in others, four groups of arachnoid plane types were differentiated, depending on the percentage of the surface area of the tumor surrounded by a clear arachnoid plane [51]. Finally, some authors noted the existence of a CSF cleft, but did not include it in their statistical analysis [53]. Surprisingly, it seems that these MRI findings do not necessarily correlate with in vitro results, since it has been reported that the thick arachnoid peritumoral capsule could actually be correlated to a larger PTBE [52].

### Irregular tumor shape

It is known that an irregular shape is not only often seen in higher-grade meningiomas [10, 32, 34, 56, 57], but also that irregularly shaped lesions can frequently recur, both in pediatric [58] and adult [32] patients. However, the connection between the irregular shape of the meningioma and PTBE seems to be less straightforward.

On one hand, many researchers found that lobulated tumors do in fact frequently exhibit large PTBE [16, 38, 41, 43, 47] (Image 1). In contrast, there have been several reports which failed to find such a connection [35, 37, 44, 46, 48] (Images 4, 6 and 7). 

These differences are probably due to the fact that the division into “regularly” and “irregularly” shaped meningiomas is mostly subjective. There are not any clear parameters to confine a meningioma into any of these groups, with the estimate instead relying entirely on the authors’ subjective assessment. Furthermore, some authors dichotomized their findings into non-lobulated versus lobulated meningiomas [16, 35, 37, 43, 44, 46–48], while others listed three distinct categories of meningioma shapes, namely round, lobulated, and mushrooming tumors [41, 52]. Finally, some lesions can appear lobulated due to surrounding structures. Whether or not such meningiomas are included in the analysis and to which group they are assigned to is explained only in rare instances [41].

### Hyperintensity and heterogeneity

Signal intensity and heterogeneity of the meningioma are also factors which seem to be connected to the lesions’ higher grade, recurrence, and/or potential to grow [10, 32–34]. They are also frequently analyzed with regard to their possible connection to PTBE. For example, it has been reported that meningiomas which are hyperintense on T2-weighted images are frequently correlated to a larger PTBE [14, 16, 43, 46, 47]. Similarly, a heterogeneous contrast enhancement on T1-weighted images is usually seen in meningiomas associated with a large PTBE [16, 48]. On the other hand, several reports did not find the connection between the extent of PTBE and either the T2 intensity of the lesion [35, 37, 53] or the pattern [14, 35] and degree [14] of its contrast enhancement (Images 7 and 8).

However, there also exist significant differences in describing and analyzing the tumor with regard to their signal intensity and heterogeneity. Lesions which appear hyperintense on T2-weighted imaging are usually thought of as having higher water content and being connected to a larger PTBE. In this way, some authors classify the meningiomas as having a low and high T2 signal intensity [47]. Others add more categories, analyzing the lesions as being hypointense, isointense, and hyperintense [35, 43]. To the hypointense or isointense lesions, some authors add another category, that of meningiomas which exhibit mixed signal intensity [53], while others add this category to three others, analyzing hypointense, isointense, hyperintense, and mixed lesions [16]. Several researchers opted for dichotomizing the signal intensity, usually into hypointense or isointense tumors as opposed to hyperintense neoplasms [14, 37, 46]. The evaluation of the contrast enhancement pattern of the lesion is also somewhat prone to different interpretations, with some authors mentioning only homogenous or heterogenous enhancement patterns [16, 35, 48] (Image 7), while others considered a lesion to be homogenously enhanced only in the absence of any intervening structures inside the tumor [14] (Image 8). Finally, there have even been proposals to classify the signal intensity on both T1- and T2-weighted images into five distinct categories each [8].

## Discussion

The differentiation of all of the aforementioned radiographic predictors of PTBE is vital, since the statistical analysis and the subsequent conclusions depend heavily on the exact methodology and differentiation of the factors analyzed. These conclusions are in turn of great clinical importance. First of all, they significantly affect the understanding of PTBE pathophysiology. In fact, radiographic analysis is the only option of analyzing meningiomas and PTBE in vivo, and as such provides valuable insight about the biology of the tumor, as well as the interaction between the brain and the neoplasm. This in turn can yield important information for future treatment, both operative and conservative. And the current knowledge about meningioma PTBE is strikingly incomplete and inconsistent. Indeed, even the conventional wisdom of how exactly PTBE is visualized radiographically has recently been brought into question [59]. Secondly, understanding radiographic appearances is paramount in the prediction of future meningioma behavior, which is in turn crucial in the analysis and risk stratification of different treatment options for meningioma patients. Recognizing the aggressive, proliferative meningiomas and differentiating them from benign and dormant ones is a crucial initial step in meningioma management. Indeed, it is well-known that the majority of meningiomas actually have a benign clinical course, with incidental and asymptomatic lesions being increasingly detected due to more readily available radiographic procedures. It has also been recognized that over-diagnoses and excessive follow-ups can be significant pitfalls in meningioma management, placing an unnecessary burden both on patients as well as the healthcare system in general [2, 5, 60]. Reliably predicting the lesion’s future behavior is therefore one of the most important initial steps of a neurosurgeon treating these patients. And the only resources at his disposal are various studies with different methodology strategies and conflicting conclusions. If the process of how and why meningioma PTBE forms remains a mystery, or in what circumstances and around which tumors PTBE develops, physicians are unfortunately bound to provide suboptimal treatment for their patients. Indeed, as seen in our article, currently it is very difficult to reliably predict meningioma behavior based on strong scientific evidence and analyzing radiographic imaging. It is therefore almost impossible to assess the risks of all treatment options during preoperative, perioperative, and postoperative care, sometimes with dire consequences for the patient.

## Conclusions

The most researched radiographic predictors of meningioma PTBE are the size of the tumor, its location, irregular margins, heterogeneity, and the peritumoral arachnoid plane with its pial vascular recruitment. In this article, we analyzed the methodology of the research conducted so far and highlighted the many controversies still present. By doing this, we believe that we have shown how little is actually known about the radiographic appearance of PTBE, which in turn has important clinical implications. It is our hope that through this article, we have emphasized the need for rigorous and uniform methodology, which can hopefully shed light on the exact nature of PTBE in meningiomas, ultimately helping and improving the lives of our patients.

## Supplementary Information

Supplementary Material 1. Image 1 Note the relatively small PTBE (red and black arrows) compared to the large size of the lesion. Note also the irregular shape of the tumor, intense and heterogenous contrast enhancement in A, and heterogenous appearance in B. (Brain MRI, axial view. A T1-weighted image with intravenous gadolinium contrast enhancement, B T2-weighted image; Pathohistology: Meningothelial Meningioma, WHO grade 1). Image 2 Note the pronounced PTBE (black arrow). Note also the round shape of the meningioma (red arrow), its relatively small size and homogenous contrast enhancement and homogenous appearance in B. (Brain MRI, axial view. A T1-weighted image with intravenous gadolinium contrast enhancement, B T2-weighted image; Pathohistology: Meningothelial Meningioma, WHO grade 1). Image 3 Note the discrete PTBE in the immediate proximity of the tumor (red arrow), as well as the several “flow voids” around the lesion, indicating peritumoral blood vessels (black arrow). Finally, note the round shape of the neoplasm, its relatively large size, as well as its adherence to the venous sinus. (Brain MRI, axial view. A T1-weighted image with intravenous gadolinium contrast enhancement, B T2-weighted image; Pathohistology: Meningothelial Meningioma, WHO grade 1). Image 4 Note the absence of any PTBE around this cerebellar meningioma (red arrow). Also note the large size and the round shape of the lesion. Finally, note the intense contrast enhancement in A, and a homogenous, hypointense appearance (compared to CSF), without any arachnoid plane or peritumoral blood vessels in B. (Brain MRI, coronal view. A T1-weighted image with intravenous gadolinium contrast enhancement, B T2-weighted image; Pathohistology: Meningothelial Meningioma, WHO grade 1). Image 5 Note the pronounced PTBE around the tumor in B. Note also the relatively small size of the lesion, its round shape as well as its homogenous contrast enhancement. Finally, note the homogenous hypointensity (compared to CSF) of the lesion in B, as well as the peritumoral hyperintense rim (red arrow) without flow voids around most of the tumor, indicating a thick arachnoid membrane between the meningioma and the brain. (Brain MRI, axial view. A T1-weighted image with intravenous gadolinium contrast enhancement, B T2-weighted image; Pathohistology: Meningothelial Meningioma, WHO grade 1). Image 6 Note the significant compression of the brain occurring due to the large size of the tumor, as well as the pronounced PTBE which occurs even in such a compressed brain. Note also the peritumoral hyperintense rim, as well as the pronounced “flow voids” (black arrows), indicating a significant peritumoral vascular supply. Finally, note the homogenous, hypointense appearance (compared to CSF) on T2-weighted imaging in B. (Brain MRI, axial view. A T1-weighted image with intravenous gadolinium contrast enhancement, B T2-weighted image; Pathohistology: Meningothelial Meningioma, WHO grade 1). Image 7 Note theabsence of a PTBE around the meningioma. Note also the lobulated, irregular shape of the tumor and the hyperintense contrast enhancement (white arrow) when compared to the venous sinus (red arrow). Finally, note the heterogenous appearance in B, as well as the peritumoral hyperintensity (black arrow), indicating a thick peritumoral arachnoid membrane. (Brain MRI, axial view. A T1-weighted image with intravenous gadolinium contrast enhancement, B T2-weighed image; Pathohistology: Meningothelial Meningioma, WHO grade 1). Image 8 Note the extensive PTBE (red arrow) around the meningioma. Note also the rounded shape of the tumor, as well as its homogenous contrast enhancement in A, and the hypointense appearance (compared to CSF) in B. Finally, note the lack of any significant peritumoral hyperintensive rim. (Brain MRI, axial view. A T1-weighted image with intravenous gadolinium contrast enhancement, B T2-weighted image; Pathohistology: Meningothelial Meningioma, WHO grade 1).

## Data Availability

All data presented in the study are available from the corresponding author upon reasonable request.
