# Supplementary material for: Radiographic predictors of peritumoral brain edema in intracranial meningiomas: a review of current controversies and illustrative cases
Source: Chin Neurosurg J. 2024 Oct 28;10:31. doi: 10.1186/s41016-024-00383-2 (PMC11514783; doi:10.1186/s41016-024-00383-2)
Supplement: Supplementary file 1 — Supplementary Material 1. Image 1 Note the relatively small PTBE (red and black arrows) compared to the large size of the lesion. Note also the irregular shape of the tumor, intense and heterogenous contrast enhancement in A, and heterogenous appearance in B. (Brain MRI, axial view. A T1-weighted image with intravenous gadolinium contrast enhancement, B T2-weighted image; Pathohistology: Meningothelial Meningioma, WHO grade 1). Image 2 Note the pronounced PTBE (black arrow). Note also the round shape of the meningioma (red arrow), its relatively small size and homogenous contrast enhancement and homogenous appearance in B. (Brain MRI, axial view. A T1-weighted image with intravenous gadolinium contrast enhancement, B T2-weighted image; Pathohistology: Meningothelial Meningioma, WHO grade 1). Image 3 Note the discrete PTBE in the immediate proximity of the tumor (red arrow), as well as the several “flow voids” around the lesion, indicating peritumoral blood vessels (black arrow). Finally, note the round shape of the neoplasm, its relatively large size, as well as its adherence to the venous sinus. (Brain MRI, axial view. A T1-weighted image with intravenous gadolinium contrast enhancement, B T2-weighted image; Pathohistology: Meningothelial Meningioma, WHO grade 1). Image 4 Note the absence of any PTBE around this cerebellar meningioma (red arrow). Also note the large size and the round shape of the lesion. Finally, note the intense contrast enhancement in A, and a homogenous, hypointense appearance (compared to CSF), without any arachnoid plane or peritumoral blood vessels in B. (Brain MRI, coronal view. A T1-weighted image with intravenous gadolinium contrast enhancement, B T2-weighted image; Pathohistology: Meningothelial Meningioma, WHO grade 1). Image 5 Note the pronounced PTBE around the tumor in B. Note also the relatively small size of the lesion, its round shape as well as its homogenous contrast enhancement. Finally, note the homogenous hypointensity (compa [file 41016_2024_383_MOESM1_ESM.docx]

**Illustrative cases**


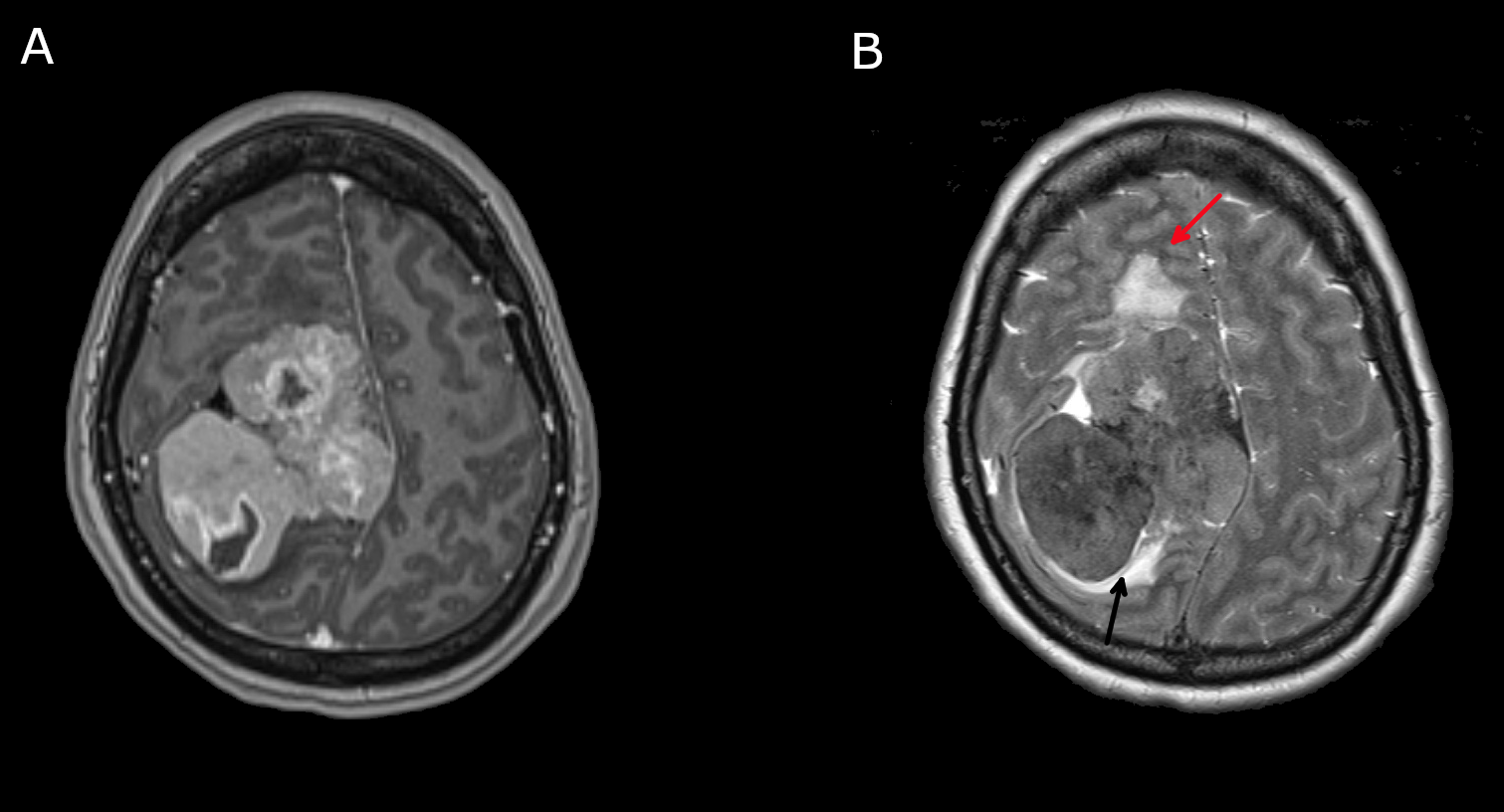


**Image 1** Note the relatively small PTBE (red and black arrows) compared to the large size of the lesion. Note also the irregular shape of the tumor, intense and heterogenous contrast enhancement in **A**, and heterogenous appearance in **B**. (Brain MRI, axial view. **A:** T1 weighed image with intravenous Gadolinium contrast enhancement, **B:** T2 weighed image; Pathohistology: Meningothelial Meningioma, WHO grade 1)


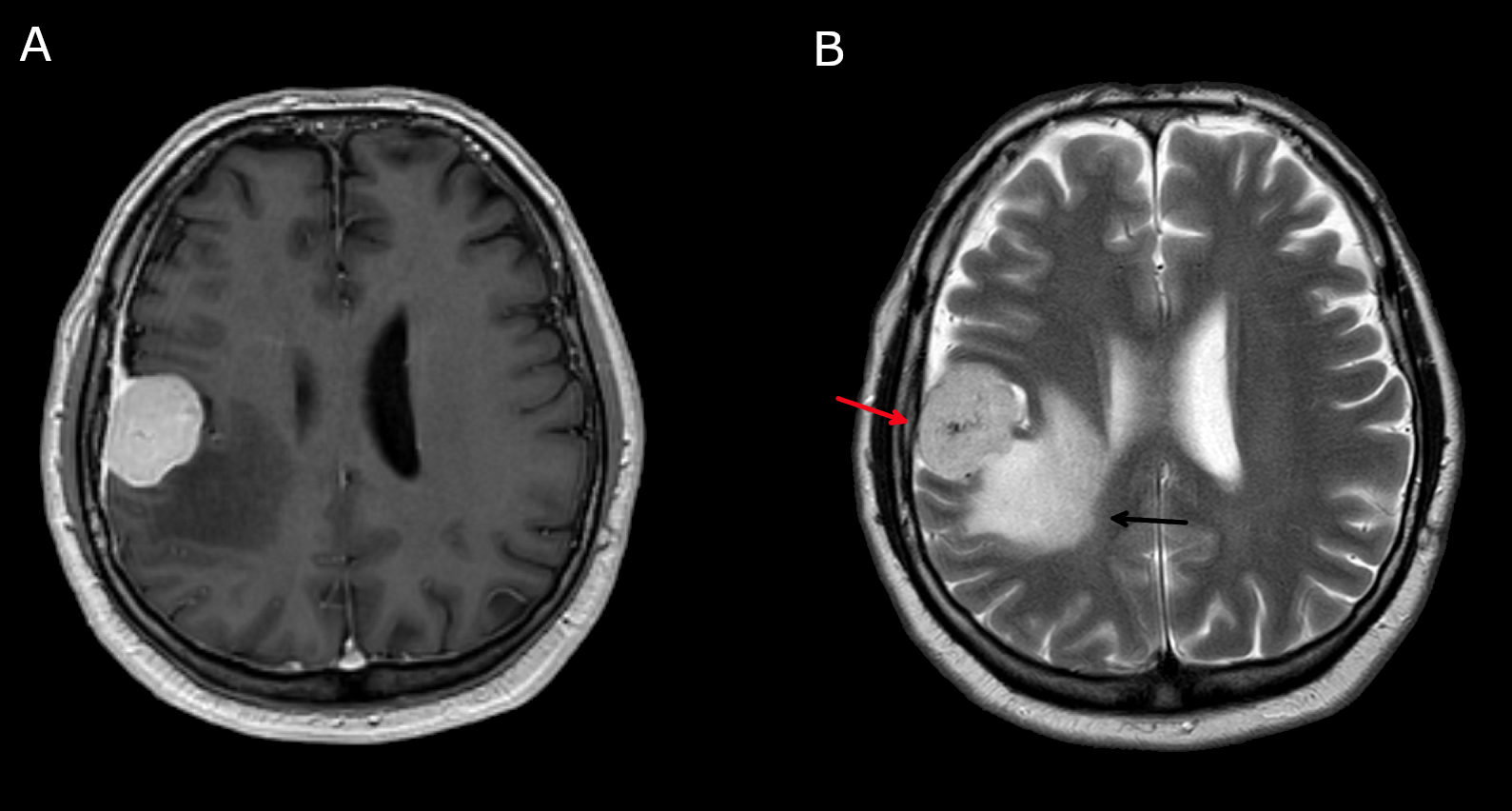


**Image 2** Note the pronounced PTBE (black arrow). Note also the round shape of the meningioma (red arrow), its’ relatively small size and homogenous contrast enhancement and homogenous appearance in **B**. (Brain MRI, axial view. **A:** T1 weighed image with intravenous Gadolinium contrast enhancement, **B:** T2 weighed image; Pathohistology: Meningothelial Meningioma, WHO grade 1)


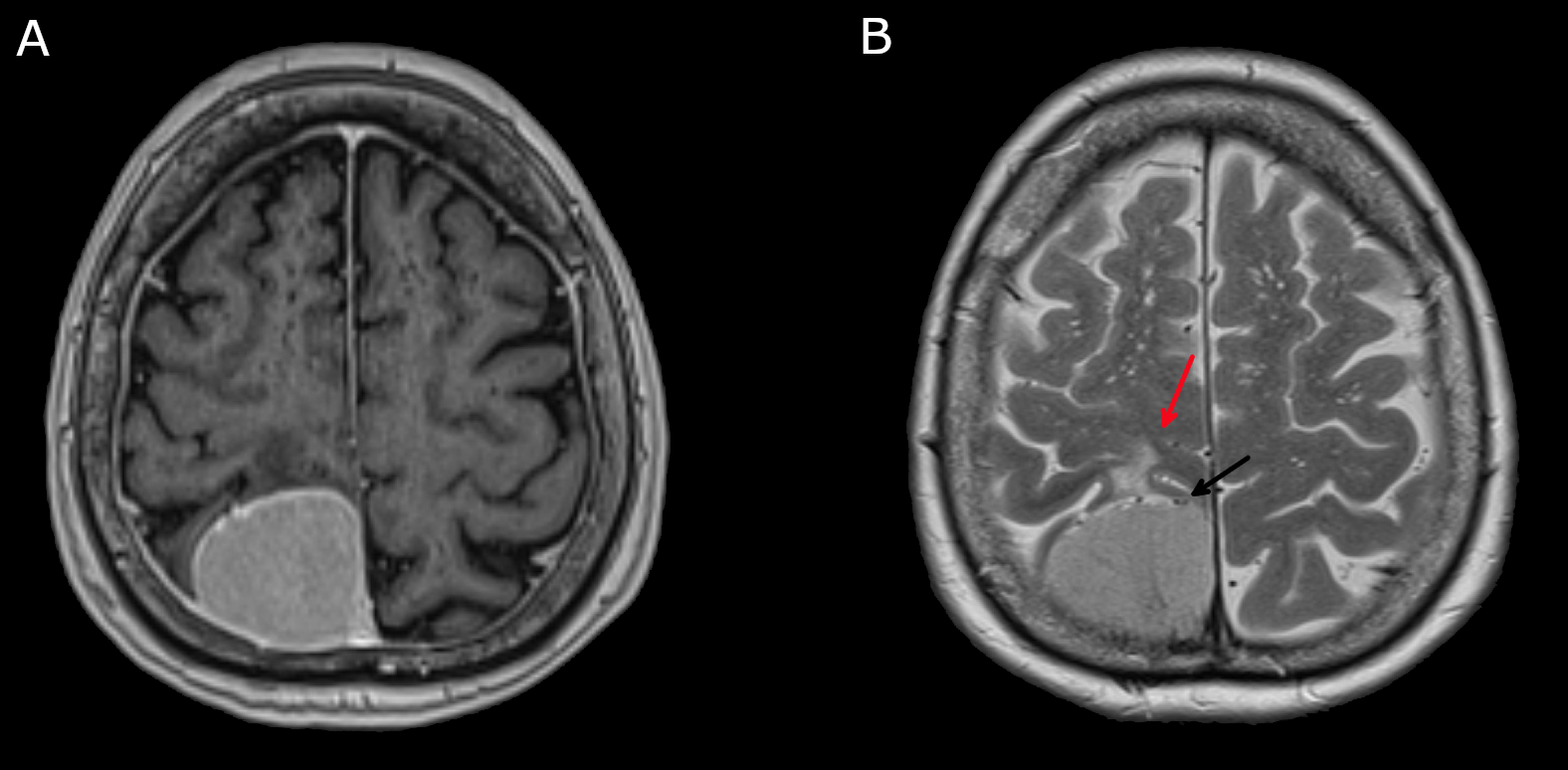


**Image 3** Note the discrete PTBE in the immediate proximity of the tumor (red arrow), as well as the several “flow voids” around the lesion, indicating peritumoral blood vessels (black arrow). Finally, note the round shape of the neoplasm, its’ relatively large size, as well as its’ adherence to the venous sinus. (Brain MRI, axial view. **A:** T1 weighed image with intravenous Gadolinium contrast enhancement, **B:** T2 weighed image; Pathohistology: Meningothelial Meningioma, WHO grade 1)


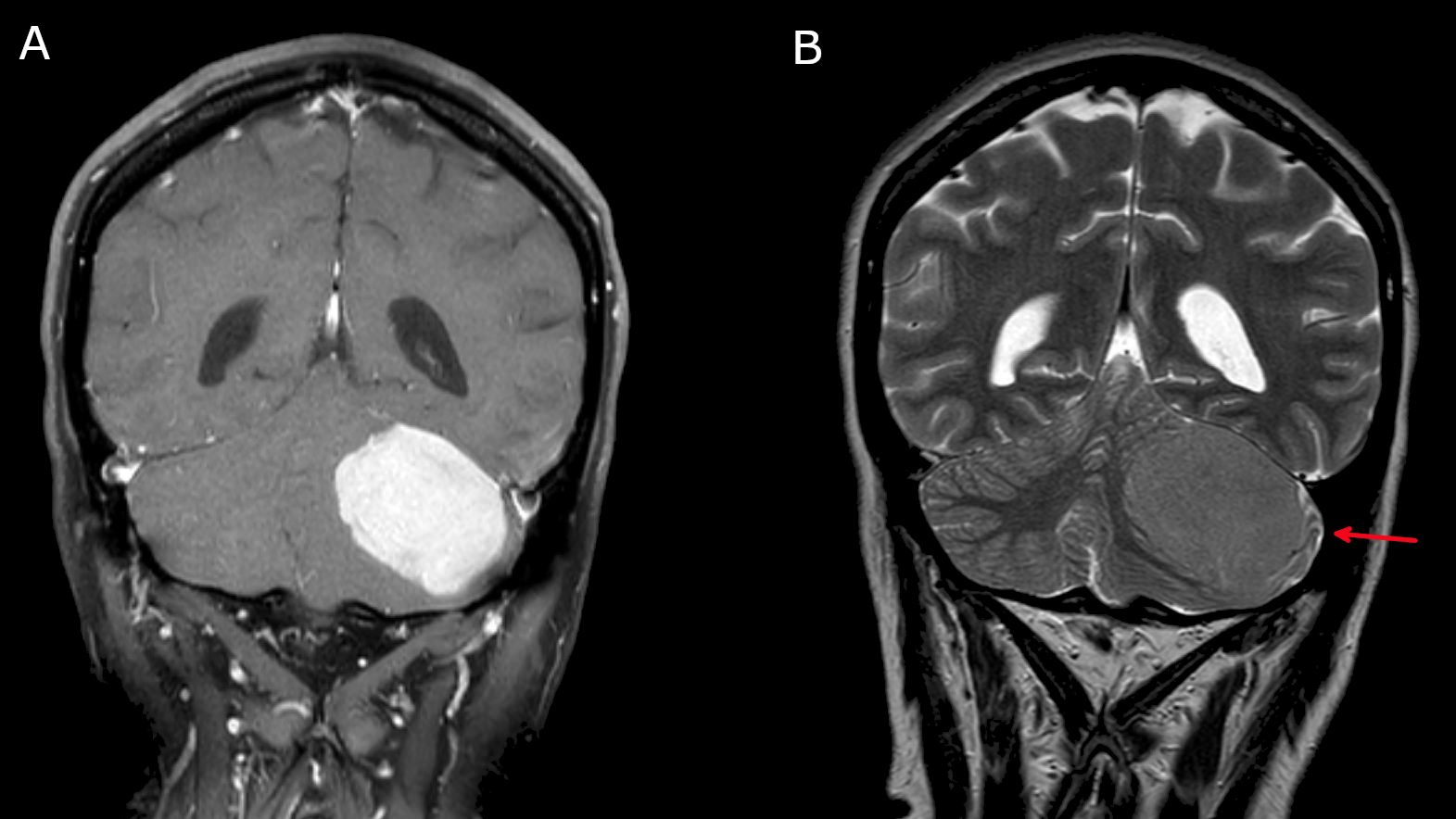


**Image 4** Note the absence of any PTBE around this cerebellar meningioma (red arrow). Also note the large size and the round shape of the lesion. Finally, note the intense contrast enhancement in **A**, and a homogenous, hypointense appearance (compared to CSF), without any arachnoid plane or peritumoral blood vessels in **B**. (Brain MRI, coronal view. **A:** T1 weighed image with intravenous Gadolinium contrast enhancement, **B:** T2 weighed image; Pathohistology: Meningothelial Meningioma, WHO grade 1)


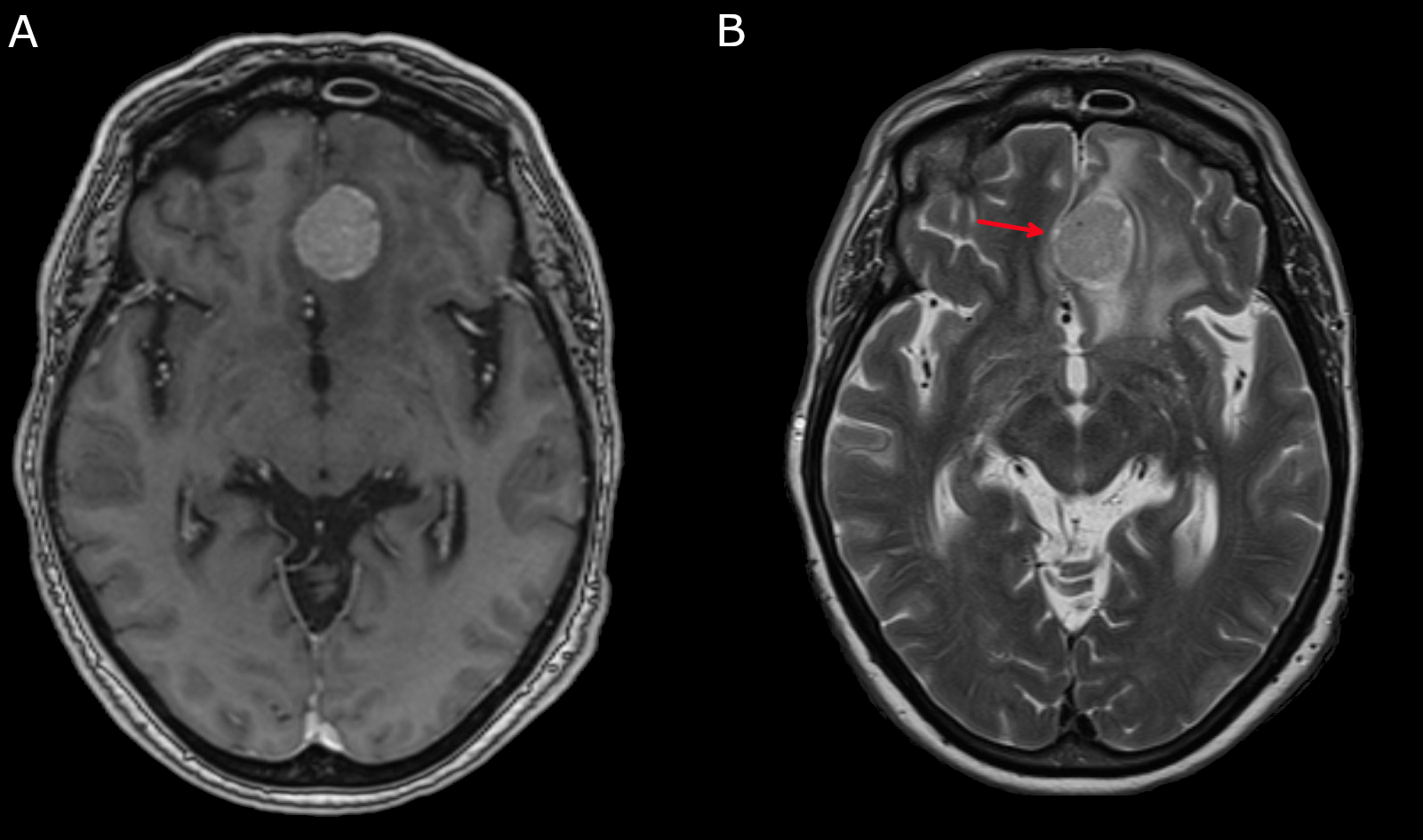


**Image 5** Note the pronounced PTBE around the tumor in **B**. Note also the relatively small size of the lesion, its’ round shape as well as its’ homogenous contrast enhancement. Finally, note the homogenous hypointensity (compared to CSF) of the lesion in **B**, as well as the peritumoral hyperintense rim (red arrow) without flow-voids around most of the tumor, indicating a thick arachnoid membrane between the meningioma and the brain. (Brain MRI, axial view. **A:** T1 weighed image with intravenous Gadolinium contrast enhancement, **B:** T2 weighed image; Pathohistology: Meningothelial Meningioma, WHO grade 1)


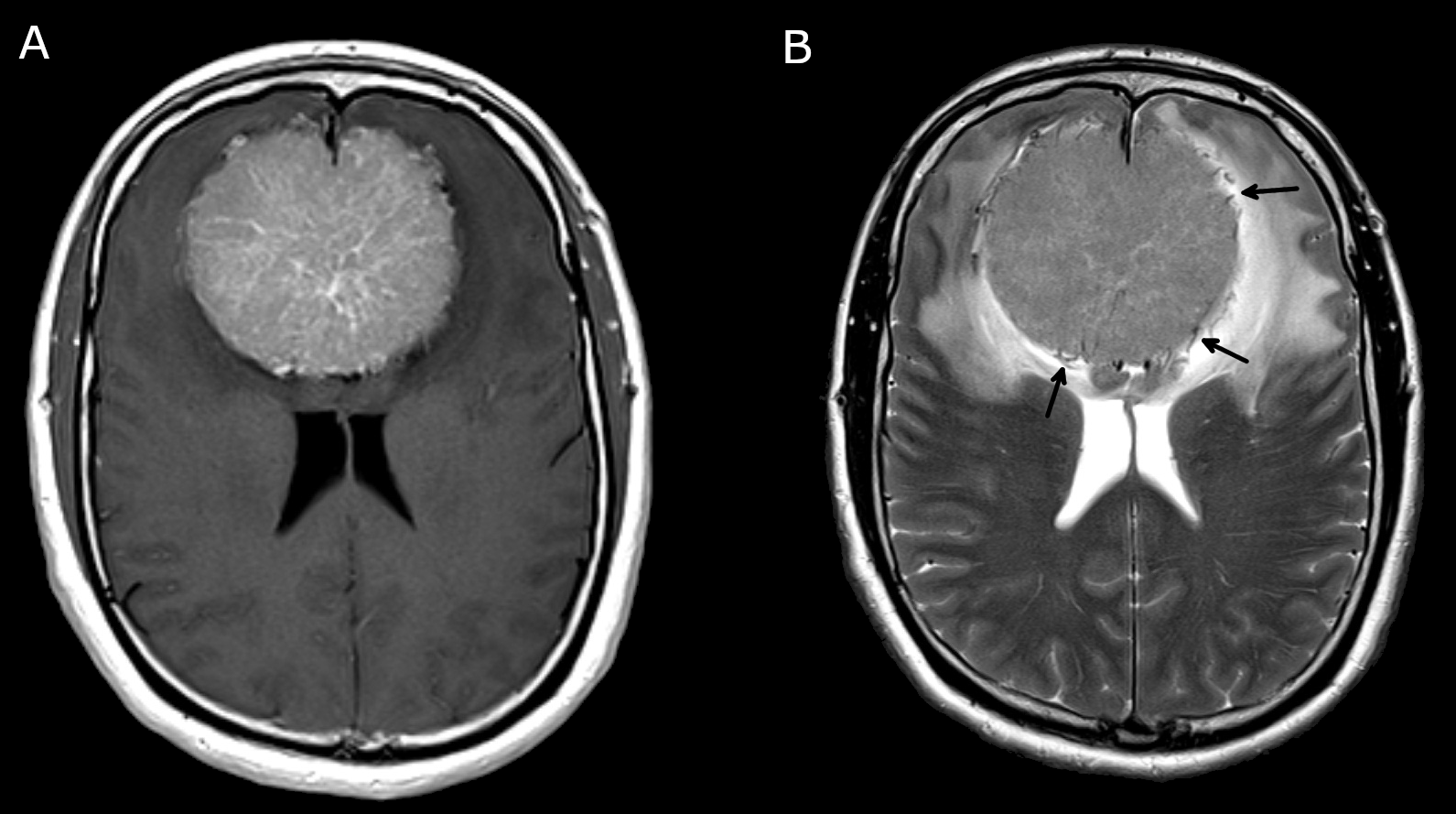


**Image 6** Note the significant compression of the brain occurring due to the large size of the tumor, as well as the pronounced PTBE which occurs even in such a compressed brain. Note also the peritumoral hyperintense rim, as well as the pronounced “flow-voids” (black arrows), indicating a significant peritumoral vascular supply. Finally, note the homogenous, hypointense appearance (compared to CSF) on T2-weighed imaging in **B**. (Brain MRI, axial view. **A:** T1 weighed image with intravenous Gadolinium contrast enhancement, **B**: T2 weighed image; Pathohistology: Meningothelial Meningioma, WHO grade 1)


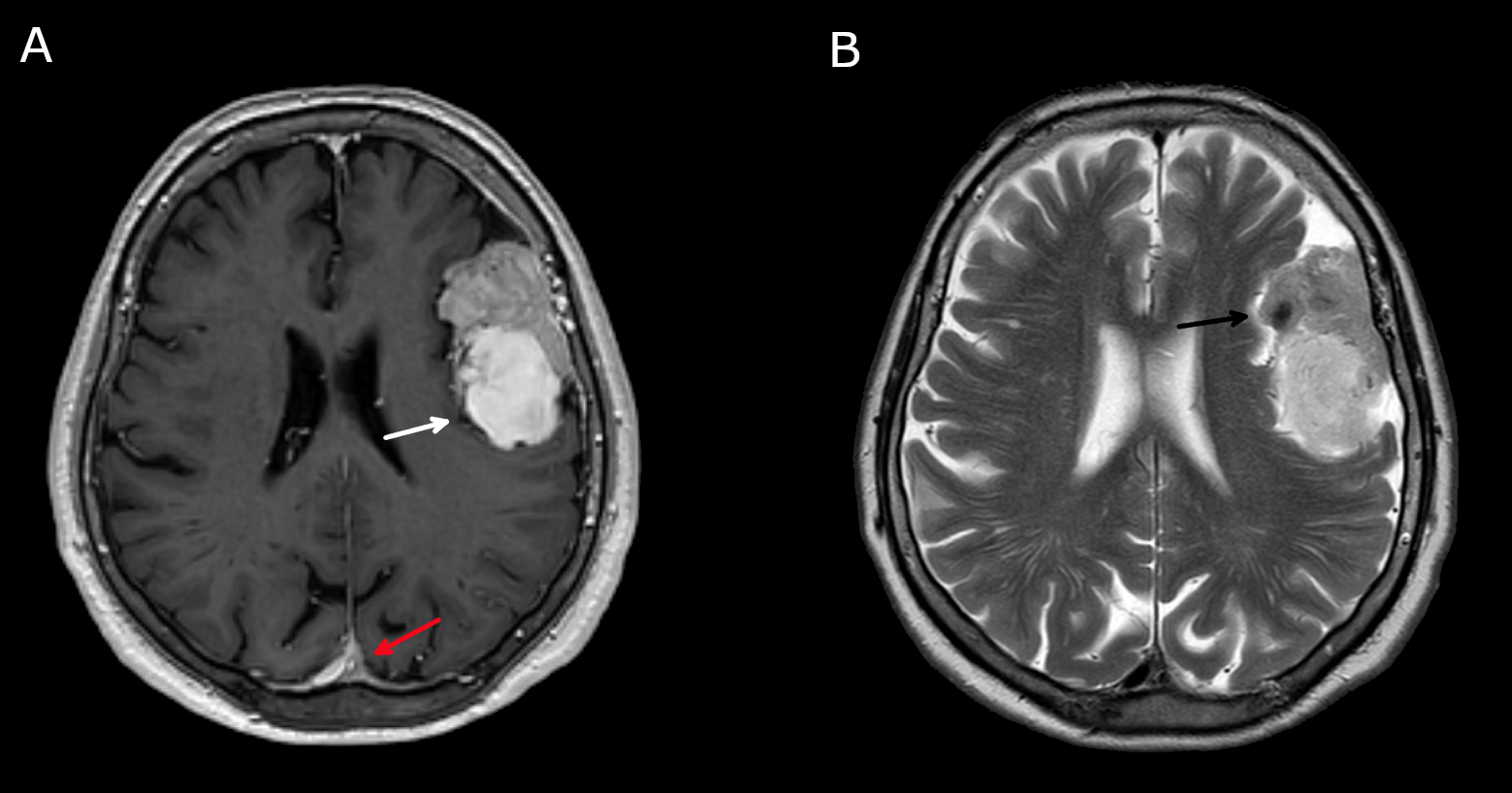


**Image 7** Note the absence of a PTBE around the meningioma. Note also the lobulated, irregular shape of the tumor and the hyperintense contrast enhancement (white arrow) when compared to the venous sinus (red arrow). Finally, note the heterogenous appearence in **B**, as well as the peritumoral hyperintensity (black arrow), indicating a thick peritumoral arachnoid membrane. (Brain MRI, axial view. **A:** T1 weighed image with intravenous Gadolinium contrast enhancement, **B:** T2 weighed image; Pathohistology: Meningothelial Meningioma, WHO grade 1)


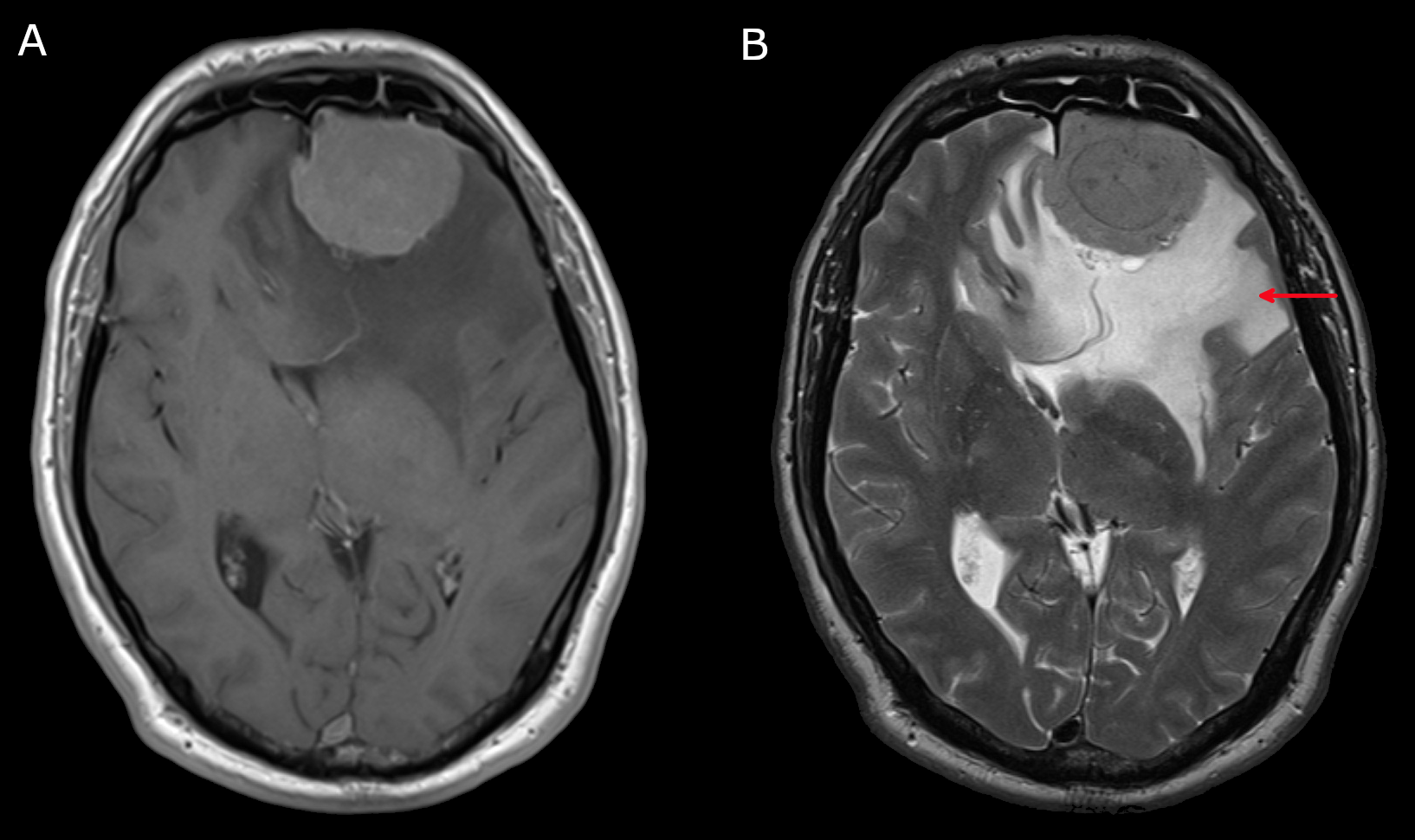


**Image 8** Note the extensive PTBE (red arrow) around the meningioma. Note also the rounded shape of the tumor, as well as its’ homogenous contrast enhancement in **A**, and the hypointense appearance (compared to CSF) in **B**. Finally, note the lack of any significant peritumoral hyperintensive rim. (Brain MRI, axial view. **A:** T1 weighed image with intravenous Gadolinium contrast enhancement, **B:** T2 weighed image; Pathohistology: Meningothelial Meningioma, WHO grade 1)
